# Case Report: Proven Diagnosis of Culture-Negative Chronic Disseminated Candidiasis in a Patient Suffering From Hematological Malignancy: Combined Application of mNGS and CFW Staining

**DOI:** 10.3389/fmed.2021.627166

**Published:** 2021-02-24

**Authors:** Yanqi Jin, Zhouhan Wang, Chunxia Zhu, Qing Yang, Yingfeng Lu, Xiaopeng Yu, Bao Hong, Xiaojing Wang, Yimin Zhang

**Affiliations:** ^1^State Key Laboratory for Diagnosis and Treatment of Infectious Diseases, National Clinical Research Center for Infectious Diseases, Collaborative Innovation Center for Diagnosis and Treatment of Infectious Diseases, The First Affiliated Hospital, College of Medicine, Zhejiang University, Hangzhou, China; ^2^Laboratory Department, The First Affiliated Hospital, College of Medicine, Zhejiang University, Hangzhou, China; ^3^Department of Infectious Diseases of Haining Campus, The First Affiliated Hospital, College of Medicine, Zhejiang University, Haining, China

**Keywords:** chronic disseminated candidiasis, metagenomics next-generation sequencing, *Candida tropicalis*, calcofluor white staining, hematological malignancies, proven diagnosis

## Abstract

Chronic disseminated candidiasis (CDC) is a severe complication with high morbidity and mortality in patients with hematological malignancies who have undergone chemotherapy. Blood or sterile liver biopsy cultures are negative due to recurrent empirical antifungal therapy. With the escalating resistance to azole-based antifungal drugs in infection by *Candida* species, pathogen identification is becoming increasingly important for determining definitive diagnosis and treatment strategy. In this case report, we present, for the first time, diagnostic confirmation of a culture-negative CDC case with *Candida tropicalis* infection using a combination of metagenomics next-generation sequencing and calcofluor white staining.

## Introduction

Chronic disseminated candidiasis (CDC; also known as “hepatosplenic candidiasis”) is a unique manifestation of invasive candidiasis. CDC carries significant morbidity and mortality in patients with hematological malignancies who have undergone chemotherapy (1–3). The previous diagnostic criteria were positive histology plus culture evidence for *Candida* species from blood or a sterile liver biopsy (4, 5). The chance of positive findings in blood cultures with an onset of the clinical and radiological picture suggestive of CDC is poor (3). The prevalence of a proven diagnosis is quite low due to the difficulty of liver puncture and recurrent use of empirical antifungal prophylaxis after chemotherapy.

Development of metagenomics next-generation sequencing (mNGS) has enabled identification of causative pathogens in infectious diseases (6) and has been applied for the diagnosis of CDC. We report a culture-negative CDC case with *Candida tropicalis* (C. tropicalis) infection diagnosed by mNGS and calcofluor white (CFW) staining.

Description of this case study was approved (2020IIT 593) by the Ethics Committee of the First Affiliated Hospital of Zhejiang University (Hangzhou, China) and by the patient.

## Case Presentation

**Figure 1:**
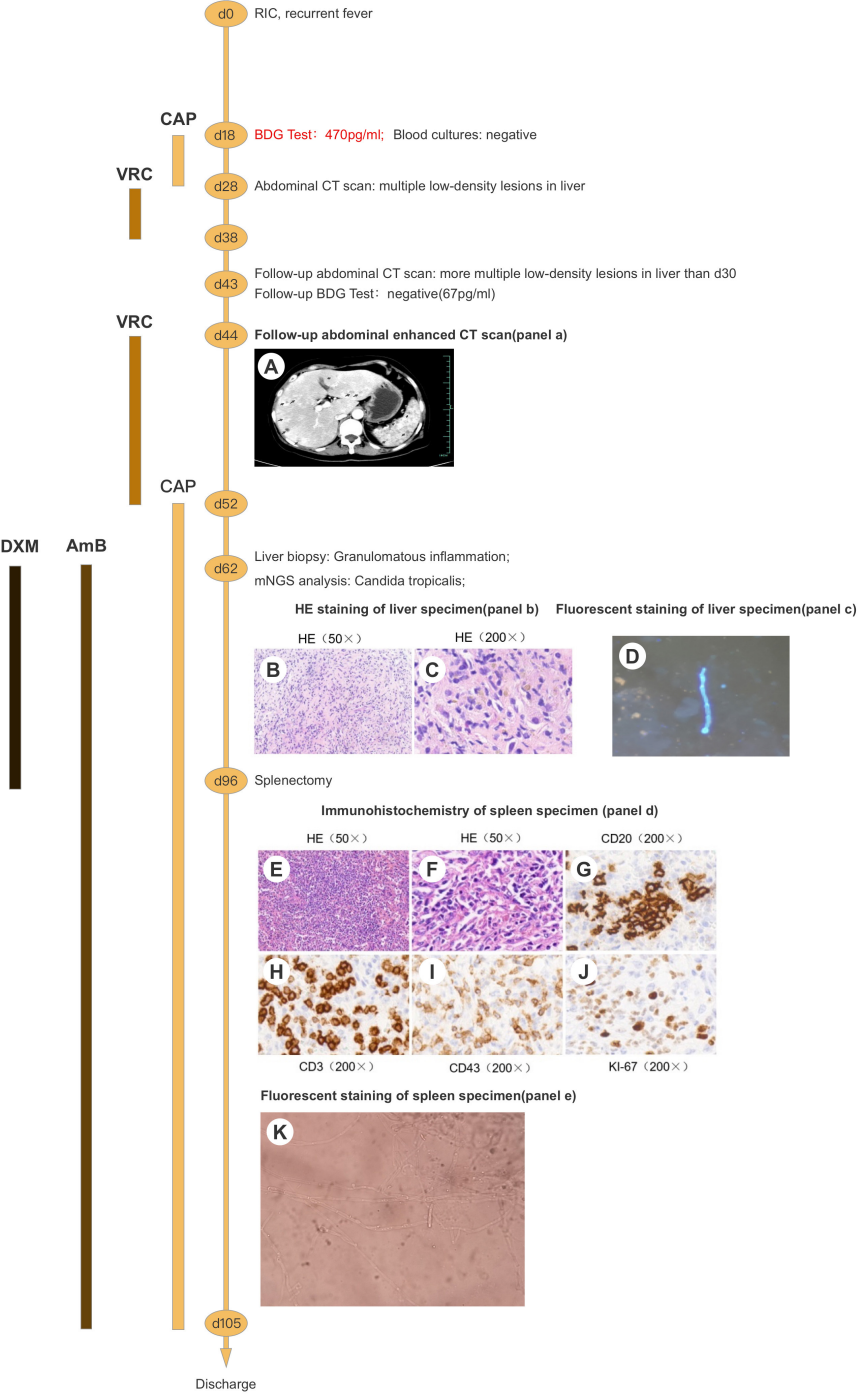
Clinical course of the patient (schematic). **(A)** Contrast-enhanced CT of the liver shows multiple nodules with marked rim-enhancement. **(B,C)** HE staining of liver specimen shows granulomatous inflammation with necrosis. **(D)** Pseudohyphae were found under fluorescence microscopy using CFW staining. **(E,F)** HE staining of spleen specimen shows granulomatous inflammation with necrosis. **(G-J)** Immunohistochemical staining of spleen specimen showed positivity for CD20 (B cells), CD3/43 (T cells), and Ki-67 (30%). **(K)** Budding cells with pseudohyphae were discovered under microscopy. RIC, remission-induction chemotherapy; BDG, (1,3)-β-D-glucan; CT, computed tomography; CAP, caspofungin; VRC, voriconazole; AmB, amphotericin B; DXM, dexamethasone; mNGS, metagenomics next-generation sequencing; HE, haematoxylin and eosin; CD, cluster of differentiation.

**Figure 2:**
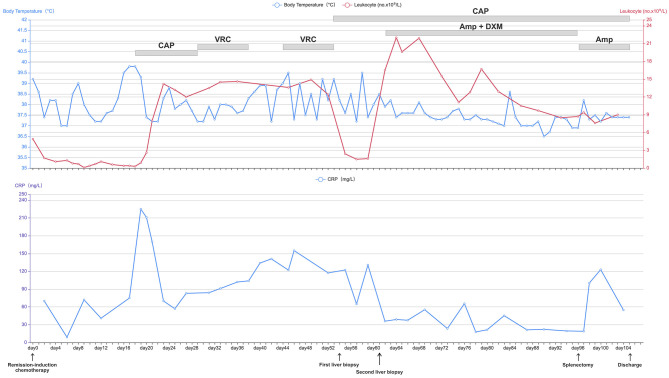
Daily course of the patient's treatment; curves of body temperature, leukocyte counts and CRP. Major events are indicated with arrows. Red line shows the leukocyte counts in peripheral blood. Blue line on the top shows body-temperature values. Blue line on the bottom shows CRP values. Horizontal thick gray lines show the medications administered: CAP, caspofungin; VRC, voriconazole; AmB, amphotericin B; DXM, dexamethasone; CRP, C-reactive protein.

A 62-year-old woman with acute myeloid leukemia-M2 received remission-induction chemotherapy (day-0). An outline of the following episodes is described in Figure 1. From day-0, recurrent fever occurred (Figure 2). Empirical treatment was undertaken with broad-spectrum antibiotics. The patient experienced neutropenia from day-2 to day-19. Antifungal prophylaxis was not employed until day-18, and yielded a positive result for the (1,3)-β-D-glucan (BDG) test (470 pg/mL). At that time, the results of a series of blood cultures were negative from day-16 to day-18 (Figure 1). Computed tomography (CT) of the abdomen showed multiple low-density lesions in the liver, spleen, and kidneys in day-28.

Caspofungin (CAP) from day-18 to day-28 and voriconazole (VRC) from day-28 to day-38 were used as antifungal therapy after a positive result for the BDG test on day-18 (Figure 1). However, the recurrent fever persisted without remission for the lesions on the liver, spleen, and kidneys even after recovery from neutropenia.

**Figure 3:**
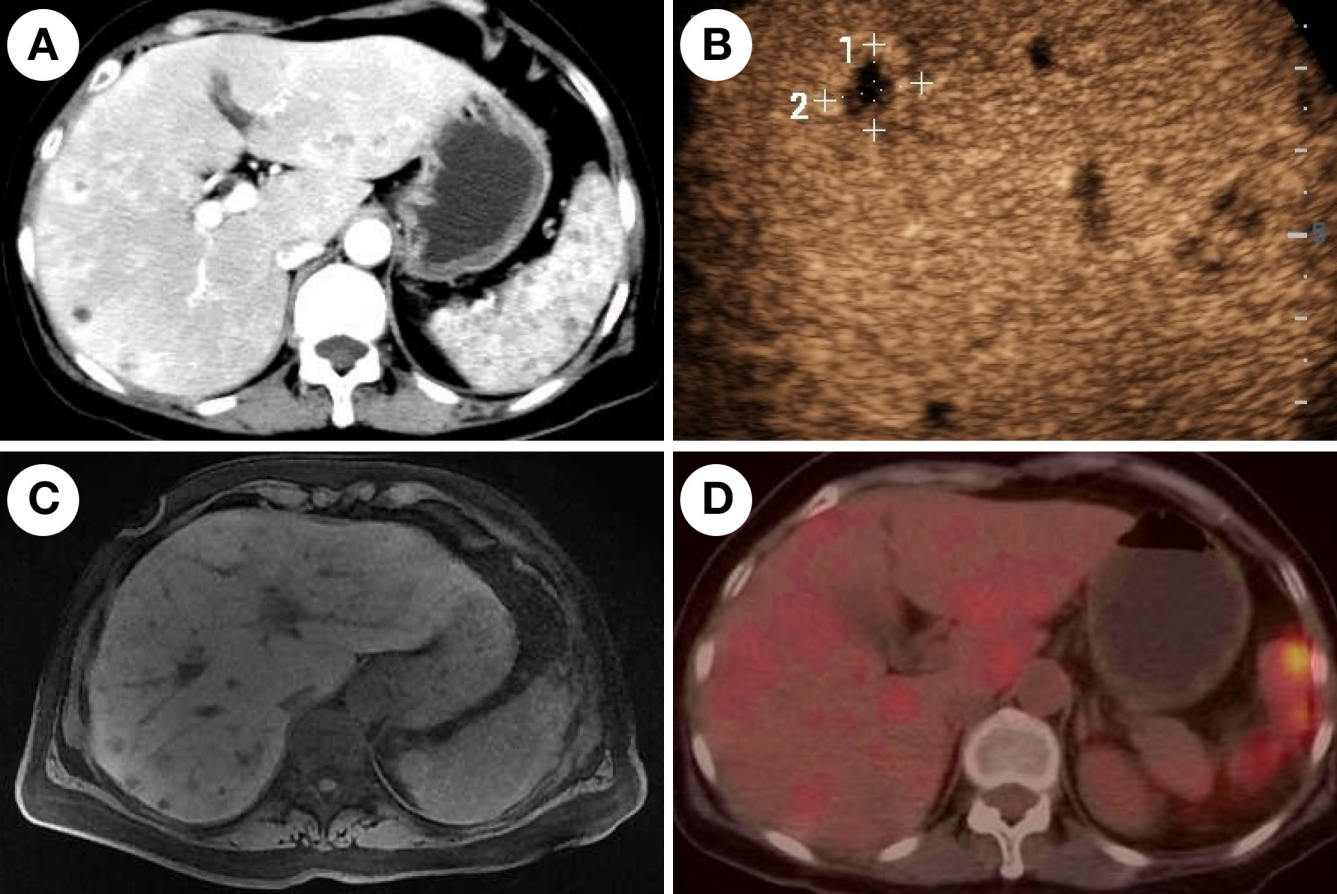
Imaging features of chronic disseminated candidiasis. **(A)** Contrast-enhanced computed tomography of the abdomen shows multiple nodules with marked rim-enhancement. **(B)** Contrast-enhanced ultrasound shows multiple hypo-echoic lesions in the liver. **(C)** Magnetic resonance imaging shows multiple-sized circular abnormal signal lesions in the liver and spleen. **(D)** ^18^F-fluorodeoxyglucose-positron emission tomography/computed tomography (^18^F-FDG-PET/CT) shows multiple lesions in the liver and spleen with increased metabolism of FDG.

Contrast-enhanced CT of the abdomen showed multiple lesions with hypodensity in the liver, spleen, and kidneys as characterized by marked rim enhancement (Figure 1A). Ultrasound of the abdomen, enhanced magnetic resonance imaging (MRI) of the abdomen, ^18^F-fluorodeoxyglucose-positron emission tomography/computed tomography, and contrast-enhanced ultrasound confirmed the result of contrast-enhanced CT of the abdomen (Figure 3). While a follow-up BDG test produced a negative result on day-43 and on several subsequent occasions (Figure 1). Expectedly, several cultures of blood and feces on different days failed to grow any pathogen.

On day-54, ultrasound-mediated liver puncture and biopsy was undertaken (Figure 2). A specimen was obtained between the boundary of lesions and normal liver tissues. Histology showed very few epithelioid cells, possibly a tumor, but no evidence of fungal infection. The antifungal agent was changed from VRC to CAP due to suspicion of CDC (Figure 2). However, her condition did not improve significantly, and recurrent fever persisted (Figure 2).

Another liver biopsy was done 1-week later (day-62). The puncture needle passed through the entire lesion to reach its center, and tissues were sampled. Histology revealed granulomatous inflammation with necrosis and no evidence of malignancy (Figures 1B,C). Immunohistochemistry showed positive in TB (FISH) (only one found) but negative in fungal (FISH). The serum T-SPOT^®^. TB interferon-γ release assay for active tuberculosis was negative. No pathogen was cultured from the two liver-biopsy tissues.

The samples from the second liver puncture were tested using mNGS (Figure 1). The results showed 55 total reads of *C. tropicalis*, accounting for 98.21% total fungal reads which was sentenced as responsible pathogen in accordance with other clinical evidences. Interestingly, pseudohyphae were found under fluorescence microscopy using CFW staining (Figure 1D) to confirm the mNGS result. After consideration of detection by mNGS and the microscopic appearance, we decided to add the antifungal agent (amphotericin B (AmB), 25 mg per day, i.v.) and adjuvant corticosteroid (dexamethasone (DXM), 7.5 mg per day, i.v.) for further treatment (Figure 2). After adjusting the medication regimen, the body temperature dropped to a normal level (Figure 2).

CAP+AmB+DXM therapy was continued for >6 weeks while patient had a normal temperature but improvement in imaging features (lesions on the liver, spleen, and kidneys) was not observed. On day-96, splenectomy was undertaken after consultation with the multidisciplinary team (Figure 1). Multiple dense abscesses were found in the entire spleen, and granulomatous inflammation with necrosis was observed in splenic tissue under microscopy (Figures 1E,F). Budding cells with pseudohyphae consistent with infection by *Candida* species were observed under microscopy (Figure 1K). Immunohistochemistry showed leukocyte common antigen and positivity for cluster of differentiation (CD)3/43 (T cells), CD20 (B cells), and Ki-67 (30%), whereas a methenamine silver stain was negative (Figures 1G–J). After splenectomy, the patient symptoms were alleviated with a decrease of CRP level and white blood cell count (Figure 2). Her body temperature remained normal and we discontinued use of DXM (Figure 2). On day-105, the patient was discharged with continuing antifungal therapy of CAP and AmB. Cytological examination of the bone marrow showed complete remission. The patient was transferred to local hospital to continue antifungal therapy and was suggested to follow-up with CT scan every 2 months.

## Discussion

The high morbidity and mortality in CDC patients necessitates rapid diagnostic. Few cases are confirmed by culture, this is supported by a retrospective single-center study from 2008 to 2013 (7). It showed very low positive results from both of blood culture (~20%) and liver biopsy(10–20%) culture from patients undergoing chemotherapy and administrated with empirical antifungal therapy (7–9).

Cultures are limited further by slow turnaround times (typically requiring 2–3 days for growth to be evident), and the fact that they often turn positive late in the course of infection (10). Hence, according to previous “gold standard” diagnostic criteria, the conventional culture-based method has limitations, and definitive treatment is often delayed (10).

MRI, CT, and ultrasound are useful for the diagnosis of CDC. However, these imaging methods do not always succeed in distinguishing fungal abscesses in the active phase from possible (even if rare) bacterial and tubercular causes (11, 12).

With the development of molecular-biology methods, diagnosis using nucleic acids in tissue was introduced in the 2020 European Organization for Research and Treatment of Cancer/Mycoses Study Group guideline of invasive fungal disease (6) compared with recommendations from the 2008 version (4). The guideline of 2020 version (6) recommended each of the following investigations as essential for the diagnosis of CDC: microscopic analyses from sterile material (including histopathologic, cytopathologic, or direct microscopic examination of a specimen); culture from sterile material; blood culture; nucleic-acid diagnosis from tissue.

A conventional method of direct microscopic examination, CFW, was introduced to the diagnosis of CDC. The *rationale* of CFW staining is to detect (non-specifically) β(1–3) and β(1–4) polysaccharides of the chitin-rich areas found in the cell walls of *Candida* species and most other fungi (13). Also, addition of CFW to preparations of potassium hydroxide can improve detection of cells from *Candida* species by increasing the contrast from background debris (14). Compared with time-consuming conventional cultures, fluorescent staining of biopsy tissues requires only ≤1 min for specimen generation and ~10 min to read samples, and is a more economical option (15). However, the disadvantage of CFW is the lack of ability to identify the species of fungus.

mNGS was an emerging culture-independent, high-throughput, microbiological diagnostic approach (16). It is relatively effective in cataloging and detecting pathogens, especially for uncommon or non-cultivable species (17). mNGS offers a faster and less biased non-culture-based methodology of pathogen detection through sequencing of extracted DNA from the sample directly (18). Therefore, mNGS has been applied to detect causal pathogens in deep-seated tissue samples (18, 19), such as in tissue from the lungs, (20), liver (21), brain (22), and cardiac-valve vegetation (23). We reported, for the first time, CDC diagnosed by mNGS. In this way, we showed that mNGS is a fast and non-culture-dependent diagnostic method.

The duration of CDC treatment can be 3–6 months (24). Usually, fluconazole is chosen after induction by CAP or AmB (25). With the escalating resistance of *Candida* species to azole (26–28), identification of *Candida* species has become important for the antifungal strategy in CDC patients. For the *C. tropicalis*, it appeared to be sensitive to fluconazole. However, drug-susceptibility testing of 20,788 strains of *Candida* species from 39 countries showed increased resistance to fluconazole for *C. tropicalis* of 2.5% in 1997 to 4.9% in 2014 (28). A survey of patients in intensive care units in China showed that the prevalence of resistance of *C. tropicalis* to fluconazole was 19.3%, but there was no resistance to CAP or AmB (29). Pathogen identification is becoming increasingly important in culture-negative CDC patients, and mNGS is a good choice.

Patients with CDC appear to occur after recovering from neutropenia, which suggests that CDC is a manifestation of immune reconstitution inflammatory syndrome (IRIS). Compared with use of an antifungal agent alone, adjuvant therapy using corticosteroids for a short course and of medium dose could benefit for patients with CDC by hastening improvement of clinical symptoms (30). Therefore, if empirical use of antifungal agents is not efficacious, identification of the pathogen in CDC is crucial to distinguish if it is caused by drug resistance or IRIS. Hence, development and validation of non-culture diagnostic methods for CDC is a top medical priority.

We combined the results from mNGS with those of CFW staining from liver biopsies. This strategy enabled confirmation of the diagnosis of CDC and guided antifungal treatment and subsequent adjuvant therapy using the corticosteroid DXM.

The histopathology manifestations of CDC are associated with three hepatic patterns: necrosis, abscess, and granuloma (31). Acute histopathology is manifested as a minimal inflammatory reaction in the neutropenic stage, whereas microabscesses with an intense inflammatory reaction occur after recovery from neutropenia. The budding cells of *Candida* species with pseudohyphae or hyphae can be seen in the center of necrotic tissue. Chronic histopathology manifests as granulomatous lesions. These are characterized by fibrosis or central necrosis surrounded by giant-cell granulomatous tissue with fibroblasts and/or macrophages arranged in palisade (9). There were only a few budding cells of *Candida* species with pseudohyphae or hyphae in the lesions in our patient. Therefore, when carrying out ultrasound-guided liver biopsy, the needle should reach the lesion center to have a greater chance of obtaining the pathogen. This strategy also explains why this specific histology feature was detected in the second liver biopsy but not in the first liver biopsy.

A combination of mNGS and CFW staining may be an alternative option for a culture-negative diagnosis of fungal infection. Nevertheless, the absence of detection of antibiotic resistance by mNGS was a limitation in this case study.

## Conclusions

mNGS testing of a liver-biopsy sample along with CFW staining is a fast and reliable method for obtaining the proven diagnosis of culture-negative CDC. Early identification of the infective pathogens in culture-negative CDC can guide pathogen-targeted antifungal chemotherapy and subsequent adjuvant therapy with corticosteroids and/or splenectomy if indicated.

## Data Availability Statement

The raw data supporting the conclusions of this article will be made available by the authors, without undue reservation.

## Ethics Statement

The studies involving human participants were reviewed and approved by Clinical Research Ethics Committee of the First Affiliated Hospital, Zhejiang University School of Medicine. The patient provided the written informed consent to participate in this study. Written informed consent was obtained from the patient for the publication of any potentially identifiable images or data included in this article.

## Author Contributions

YZ designed research. YJ, ZW, and YZ analyzed and collected data, and wrote the manuscript. CZ, BH, XW, QY, YL, and XY collected clinical data. All authors read and approved the final manuscript.

## Conflict of Interest

The authors declare that the research was conducted in the absence of any commercial or financial relationships that could be construed as a potential conflict of interest.
